# Supplementary material for: Unveiling the Bioactive Potential of the Invasive Jellyfish Phyllorhiza punctata Through Integrative Transcriptomic and Proteomic Analyses
Source: Biomolecules. 2025 Aug 4;15(8):1121. doi: 10.3390/biom15081121 (PMC12383608; doi:10.3390/biom15081121)
Supplement: Supplementary file 1 [file biomolecules-15-01121-s001.zip › Suplementary Material/FileS3/PEPFOLD4/CAH3146556_8481/clusters.html]

 PEP-FOLD clusters


Download archive of all models  here   

|  |  |  |
| --- | --- | --- |
| # | file | sOPEP |
| # cluster 1 - Size: 15 |  |  |
|  | CAH3146556\_8481-model1 | -16.6168 |
|  | CAH3146556\_8481-model1.1 | -16.5743 |
|  | CAH3146556\_8481-model1.2 | -16.5687 |
|  | CAH3146556\_8481-model1.3 | -16.536 |
|  | CAH3146556\_8481-model1.4 | -16.536 |
|  | CAH3146556\_8481-model1.5 | -16.536 |
|  | CAH3146556\_8481-model1.6 | -16.536 |
|  | CAH3146556\_8481-model1.7 | -16.5124 |
|  | CAH3146556\_8481-model1.8 | -16.4472 |
|  | CAH3146556\_8481-model1.9 | -16.4312 |
|  | CAH3146556\_8481-model1.10 | -16.4311 |
|  | CAH3146556\_8481-model1.11 | -16.4311 |
|  | CAH3146556\_8481-model1.12 | -16.3894 |
|  | CAH3146556\_8481-model1.13 | -16.3739 |
|  | CAH3146556\_8481-model1.14 | -16.3229 |
| # cluster 2 - Size: 13 |  |  |
|  | CAH3146556\_8481-model2 | -16.6059 |
|  | CAH3146556\_8481-model2.1 | -16.6059 |
|  | CAH3146556\_8481-model2.2 | -16.6059 |
|  | CAH3146556\_8481-model2.3 | -16.6059 |
|  | CAH3146556\_8481-model2.4 | -16.6059 |
|  | CAH3146556\_8481-model2.5 | -16.5097 |
|  | CAH3146556\_8481-model2.6 | -16.5097 |
|  | CAH3146556\_8481-model2.7 | -16.5097 |
|  | CAH3146556\_8481-model2.8 | -16.5097 |
|  | CAH3146556\_8481-model2.9 | -16.4418 |
|  | CAH3146556\_8481-model2.10 | -16.4265 |
|  | CAH3146556\_8481-model2.11 | -16.4265 |
|  | CAH3146556\_8481-model2.12 | -16.38 |
| # cluster 3 - Size: 20 |  |  |
|  | CAH3146556\_8481-model3 | -16.5861 |
|  | CAH3146556\_8481-model3.1 | -16.5861 |
|  | CAH3146556\_8481-model3.2 | -16.5861 |
|  | CAH3146556\_8481-model3.3 | -16.5861 |
|  | CAH3146556\_8481-model3.4 | -16.5861 |
|  | CAH3146556\_8481-model3.5 | -16.4758 |
|  | CAH3146556\_8481-model3.6 | -16.4758 |
|  | CAH3146556\_8481-model3.7 | -16.4758 |
|  | CAH3146556\_8481-model3.8 | -16.4758 |
|  | CAH3146556\_8481-model3.9 | -16.4758 |
|  | CAH3146556\_8481-model3.10 | -16.4758 |
|  | CAH3146556\_8481-model3.11 | -16.4758 |
|  | CAH3146556\_8481-model3.12 | -16.4758 |
|  | CAH3146556\_8481-model3.13 | -16.4758 |
|  | CAH3146556\_8481-model3.14 | -16.4758 |
|  | CAH3146556\_8481-model3.15 | -16.3964 |
|  | CAH3146556\_8481-model3.16 | -16.3737 |
|  | CAH3146556\_8481-model3.17 | -16.3737 |
|  | CAH3146556\_8481-model3.18 | -16.3646 |
|  | CAH3146556\_8481-model3.19 | -16.2832 |
| # cluster 4 - Size: 17 |  |  |
|  | CAH3146556\_8481-model4 | -16.5558 |
|  | CAH3146556\_8481-model4.1 | -16.5558 |
|  | CAH3146556\_8481-model4.2 | -16.5558 |
|  | CAH3146556\_8481-model4.3 | -16.5379 |
|  | CAH3146556\_8481-model4.4 | -16.5116 |
|  | CAH3146556\_8481-model4.5 | -16.5116 |
|  | CAH3146556\_8481-model4.6 | -16.4518 |
|  | CAH3146556\_8481-model4.7 | -16.4456 |
|  | CAH3146556\_8481-model4.8 | -16.44 |
|  | CAH3146556\_8481-model4.9 | -16.4071 |
|  | CAH3146556\_8481-model4.10 | -16.3871 |
|  | CAH3146556\_8481-model4.11 | -16.3871 |
|  | CAH3146556\_8481-model4.12 | -16.3871 |
|  | CAH3146556\_8481-model4.13 | -16.3871 |
|  | CAH3146556\_8481-model4.14 | -16.3871 |
|  | CAH3146556\_8481-model4.15 | -16.3013 |
|  | CAH3146556\_8481-model4.16 | -16.3013 |
| # cluster 5 - Size: 9 |  |  |
|  | CAH3146556\_8481-model5 | -16.5055 |
|  | CAH3146556\_8481-model5.1 | -16.4224 |
|  | CAH3146556\_8481-model5.2 | -16.4166 |
|  | CAH3146556\_8481-model5.3 | -16.4166 |
|  | CAH3146556\_8481-model5.4 | -16.4166 |
|  | CAH3146556\_8481-model5.5 | -16.3776 |
|  | CAH3146556\_8481-model5.6 | -16.3573 |
|  | CAH3146556\_8481-model5.7 | -16.2823 |
|  | CAH3146556\_8481-model5.8 | -16.2823 |
| # cluster 6 - Size: 1 |  |  |
|  | CAH3146556\_8481-model6 | -16.4658 |
| # cluster 7 - Size: 9 |  |  |
|  | CAH3146556\_8481-model7 | -16.4619 |
|  | CAH3146556\_8481-model7.1 | -16.4619 |
|  | CAH3146556\_8481-model7.2 | -16.4619 |
|  | CAH3146556\_8481-model7.3 | -16.4619 |
|  | CAH3146556\_8481-model7.4 | -16.4541 |
|  | CAH3146556\_8481-model7.5 | -16.4541 |
|  | CAH3146556\_8481-model7.6 | -16.4541 |
|  | CAH3146556\_8481-model7.7 | -16.4541 |
|  | CAH3146556\_8481-model7.8 | -16.3723 |
| # cluster 8 - Size: 4 |  |  |
|  | CAH3146556\_8481-model8 | -16.411 |
|  | CAH3146556\_8481-model8.1 | -16.411 |
|  | CAH3146556\_8481-model8.2 | -16.3974 |
|  | CAH3146556\_8481-model8.3 | -16.302 |
| # cluster 9 - Size: 1 |  |  |
|  | CAH3146556\_8481-model9 | -16.3835 |
| # cluster 10 - Size: 7 |  |  |
|  | CAH3146556\_8481-model10 | -16.3661 |
|  | CAH3146556\_8481-model10.1 | -16.3661 |
|  | CAH3146556\_8481-model10.2 | -16.3661 |
|  | CAH3146556\_8481-model10.3 | -16.3661 |
|  | CAH3146556\_8481-model10.4 | -16.3661 |
|  | CAH3146556\_8481-model10.5 | -16.3661 |
|  | CAH3146556\_8481-model10.6 | -16.206 |
| # cluster 11 - Size: 1 |  |  |
|  | CAH3146556\_8481-model11 | -16.3461 |
| # cluster 12 - Size: 3 |  |  |
|  | CAH3146556\_8481-model12 | -16.3361 |
|  | CAH3146556\_8481-model12.1 | -16.3226 |
|  | CAH3146556\_8481-model12.2 | -16.2733 |
|  |  |  |
